# Microeconomic institutions and personnel economics for health care delivery: a formal exploration of what matters to health workers in Rwanda

**DOI:** 10.1186/s12960-017-0261-9

**Published:** 2018-01-26

**Authors:** Pieter Serneels, Tomas Lievens

**Affiliations:** 1 University of East Anglia, Norwich, United Kingdom; 2 Oxford Policy Management, Oxford, United Kingdom

## Abstract

**Background:**

Most developing countries face important challenges regarding the quality of health care, and there is a growing consensus that health workers play a key role in this process. Our understanding as to what are the key institutional challenges in human resources, and their underlying driving forces, is more limited. A conceptual framework that structures existing insights and provides concrete directions for policymaking is also missing.

**Methods:**

To gain a bottom-up perspective, we gather qualitative data through semi-structured interviews with different levels of health workers and users of health services in rural and urban Rwanda. We conducted discussions with 48 health workers and 25 users of health services in nine different groups in 2005. We maximized within-group heterogeneity by selecting participants using specific criteria that affect health worker performance and career choice. The discussion were analysed electronically, to identify key themes and insights, and are documented with a descriptive quantitative analysis relating to the associations between quotations. The findings from this research are then revisited 10 years later making use of detailed follow-up studies that have been carried out since then.

**Results:**

The original discussions identified both key challenges in human resources for health and driving forces of these challenges, as well as possible solutions. Two sets of issues were highlighted: those related to the size and distribution of the workforce and those related to health workers’ on-the-job performance. Among the latter, four categories were identified: health workers’ poor attitudes towards patients, absenteeism, corruption and embezzlement and lack of medical skills among some categories of health workers. The discussion suggest that four components constitute the deeper causal factors, which are, ranked in order of ease of malleability, incentives, monitoring arrangements, professional and workplace norms and intrinsic motivation. Three institutional innovations are identified that aim at improving performance: performance pay, community health workers and increased attention to training of health workers. Revisiting the findings from this primary research making use of later in-depth studies, the analysis demonstrates their continued relevance and usefulness. We discuss how the different factors affect the quality of care by impacting on health worker performance and labour market choices, making use of insights from economics and development studies on the role of institutions.

**Conclusion:**

The study results indicate that health care quality to an important degree depends on four institutional factors at the microlevel that strongly impact on health workers’ performance and career choice, and which deserve more attention in applied research and policy reform. The analysis also helps to identify ways forwards, which fit well with the Ministry’s most recent strategic plan.

## Background

Most developing countries face considerable challenges regarding the quality of health care. There is an increasing consensus that many of the problems have to do with both the performance and career choices of health workers, as they form the foundation of health service delivery. Issues like health worker absenteeism, poor attitudes towards patients, shirking and embezzlement have been well documented across a number of contexts and countries.^1^ But although recent work in development thinking emphasizes a central role of institutions, generally defined as the ‘rules of the game’,^2^ surprisingly little work focuses on microeconomic institutional factors for health service delivery, in particular those related to human resources, which is the focus of this paper.^3^ The study’s aim is to explore which institutional factors may help explain the (often disappointing) performance of health workers, who actively respond to the work environment and incentives they face.

One potential reason for the scarcity of work in this area is that it is unclear what the relevant theoretical framework should be. Under these circumstances, exploratory analysis provides a useful starting point, to identify the problems and characterize the institutional factors that may offer explanations, as also suggested by Mookherjee [70]. This can then serve as a base for the formulation of theory and hypotheses.

The core of this paper is based on findings from primary qualitative research, which has been successfully applied for this purpose across disciplines, especially in medical and anthropological research. We conducted semi-structured group discussions with health workers and users of health services in both rural and urban areas in Rwanda in 2005.

Despite substantial economic growth and significant financial resources allocated to the health sector, Rwanda’s health outcomes remained poor and below target; this was the case in 2005, at the time of the data collection, and remains the case today. Major health indicators including infant, child and maternal mortality as well as life expectancy at birth have remained below the Sub-Sahara African average [106]. The Government of Rwanda did major efforts to rebuild the health system, and this resulted in important improvements in health infrastructure. Nevertheless, the utilization of health services remained low at 38%, with a declining trend in utilization between 1997 and 2003 [62]. This low utilization occurred despite a high population density—a stark contrast with most Sub-Sahara African countries—and a traditionally mixed public-private health sector, which could work in favour of utilization. Analysts agree that human resources for health formed the main explanation for this poor performance and a key constraint for better health service delivery in Rwanda. With respect to workforce size, Rwanda had a low health worker to population ratio, even by Sub-Sahara African standards: for eight out of nine staff categories, Rwanda scores below the regional average.^4^ Community health workers are the only exception, as Rwanda has comparatively more of them. Like most countries, Rwanda also faced a shortage of health workers in rural areas, even though these areas could be reached relatively well. In 2006, only 17% of health workers in the public sector took up a job in a rural area [63].

Since the early 2000s, Rwanda accelerated the implementation of important changes in the health sector. The fieldwork for the first phase of this research was implemented while these changes took place, giving us a unique perspective on the impact of these perceived transitions according to both users and health workers themselves. In a second phase of our research, we then revisit the findings of this first phase 10 years later.

In the next section, we describe the methodology and data, while the ‘Findings from primary qualitative research 2005’ section discusses the results from the group discussions. The ‘Revisiting the findings in 2015’ section provides findings from the second phase of the research, while the ‘Discussion’ section provides a discussion of the findings, and the ‘Conclusion’ section concludes.

## Methods and data

Qualitative research offers strong tools for exploratory analysis. Group discussions in particular are useful when the research wants to reveal and contrast a multitude of perceptions and opinions and have been used with success in both medical and market research.^5^ The factors described in this paper emerged from analysing the qualitative data we obtained from semi-structured group discussions with health workers and users of health services in Rwanda. Following a strict methodology in the preparation and implementation, we held discussions with 73 individuals, 48 health workers and 25 users of health services. We held six separate group discussions with different levels of health workers—doctors, nurses and auxiliary health workers, half of them in an urban area, the other half in a rural town—and three group discussions with users: one in an urban area, one in a provincial town and one separate discussion with Persons Living with HIV/AIDS (PLWHA). To allow for a richer exploration, we maximized within-group heterogeneity by selecting the participants using a number of criteria that affect performance and career choice, like age, gender, having children or not, the sector of activity (private for profit, private not-for-profit, public), the type of facility (hospital, clinic, health post), having multiple jobs or not and working at a facility with a performance initiative. Table 1 presents the group and individual profile of the participants.^6^ The groups are overall balanced in terms of gender and in terms of the number of children participants have. Health workers are predominantly active in the public sector which both reflect reality and the focus of this study. Most of them supervise other personnel active in the health facility.

**Table 1:** Discussion groups and participants

		Kigali	Provincial town	Total	Percent of total
Focus groups		5	4	9	100
Auxiliary nurses (A3 and A4)		1	1	2	22
Nurses (A1 and A2)		1	1	2	22
Doctors (A0)		1	1	2	22
Health users		1	1	2	22
Persons living with HIV and AIDS		1	0	1	11
Health workers		24	24	48	100
Of which:	Auxiliary nurses	8	8	16	33
	Nurses	8	8	16	33
	Doctors	8	8	16	33
	Female	17	8	25	52
	With children	22	19	41	85
	Below age 36	15	10	25	52
	Public	18	20	38	79
	Private for profit	13	2	15	31
	NGO	1	2	3	6
	Has formal second job	3	2	5	10
	With performance initiative	11	16	27	56
Users		17	8	25	100
Of which:	Persons living with HIV/AIDS	9	0	9	36
	Other patients/users	8	8	16	56
	Female	10	3	13	52
	With children	17	8	25	100
	Last visit in health centre	7	7	14	56
	Last visit in hospital	10	1	11	44
	Last visit in public sector facility	13	5	18	72
	Last visit in private for profit sector facility	2	0	2	8
	Last visit in NGO/faith-based facility	2	3	5	20

This first part of the study was implemented in November 2005, and the discussions took place in a meeting room in a health facility, were semi-structured using detailed scripts, lasted on average 2 h and a half and were held in French for the doctors, nurses and pharmacists and in Kinyarwanda for the auxiliary health workers and the users.^7^ All discussions were recorded and transcribed in French, which was the second national language at the time, together with Kinyarwanda.

The transcripts were then coded and subsequently analysed following the four steps set out below. During the coding, a label was attributed to each unit of text or quotation, reflecting the underlying content of the topic discussed. A quotation can be given different codes when it refers to different topics at the same time. Where this occurs, the association of codes can be analysed. In total, 1203 quotes have been examined and 35 codes have been used, reflecting the diversity of the issues of interest in the study. The text was then analysed with QSR NVIVO, to identify emerging key themes and insights.

The analysis is carried out in four steps. In a first step, all quotes by participants are grouped in more or less homogeneous groups, called ‘free nodes’; no pre-established node-structure is used. In total, 27 free nodes were identified; some examples are ‘performance evaluation’, ‘remuneration’ and ‘performance pay’. In a second step, logical relations between free nodes were identified, creating a node tree-structure. A third step further explores relationships between free nodes, for example, verifying whether participants make reference to ‘performance pay’ when talking about ‘performance evaluation’ to examine relationships that might not be explicitly apparent. Finally, we compare the number of quotes per topic. A large number of quotations is seen as suggestive of the relative importance of that topic to health workers. A low number of quotations corresponds to the situation where the interviewer has prompted health workers about a topic but gets very few replies because the topic is not an issue. The analysis then focused on those issues with the largest number of mentions. Three categories of themes emerged: problems and challenges, key explanatory factors and ongoing and planned innovations. The nature of the explanatory factors that received most attention alerted the researchers to the overarching role of institutions, which is put forward as the appropriate conceptual framework to interpret the findings. The quotes reported in this paper are selected because of their salience and because they reflect themes and issues recurrently brought up by participants.

In a second phase, we revisit the findings from this primary research 10 years later relying on several in-depth studies that have taken place in Rwanda since then, as well as official documents from the Government of Rwanda. The studies were primarily identified through search of Google Scholar, PubMed and Econlit and databases of PhD theses. Other ongoing research, research under review and policy reports were identified through google search and through consultation of experts on health care in Rwanda.

The data limitations are twofold. The choice for group discussions in the first phase, while deliberate in order to generate a broad set of insights, comes at the expense of being able to carry out analysis at the level of individual health worker. Second, the revisit of the findings from the first phase makes use of a different method, using insights from existing studies. This allows for a richer comparison and evaluation in some ways but also has a cost; for instance, it does not allow for consistent comparison across these same respondent groups.

## Findings from primary qualitative research 2005

The discussions centre around two big clusters of insights: those to do with identification of health worker problems and challenges and those related to possible solutions to these problems. We discuss each in turn.

### Problems and challenges?

What are the challenges with human resources for health in Rwanda? It emerges from the discussions that these can be divided into those related to the size and distribution of the workforce and those associated with health workers’ on-the-job performance.

#### Size and distribution of the workforce

Health worker participants in the discussions highlight the existing shortage in number of health workers but also point to shortcomings in human resource practices and education policies that exacerbate the situation. Facilities that have been mandated to recruit staff directly often seem to lack the resources to do so. As a consequence, especially junior nurses often have to wait before they can take up their first job. On the education side, it is a mystery to most health workers why there is still a cap on entry into post-secondary medical education (‘numerus clausus’), while the limited access to secondary education in general restricts the number of students who can enrol for either nursing or medical studies.

Since health workers can express a preference where to work, the least popular positions tend to be understaffed. This is especially the case for higher educated health workers who are less numerous and can therefore be more picky about their place of work. One example is the shortage of health workers in rural areas. When health worker participants talk about frustration and dissatisfaction in the profession, they frequently relate this to rural postings (15% of the cases). Private sector positions are often more popular than public sector ones, while jobs in public health seem to be more popular than those in clinical care, because they are often better paid, but also because they involve no health risks. When a job compensates for these kinds of risks, it manages to attract health workers. The discussions suggest that higher earnings contribute to the greater popularity of private sector jobs: when health workers discuss high pay, in 15 out of the 17 cases, they refer to the private sector, for-profit or NGO’s, and often mention HIV/AIDS services.

While shortages in some positions—for instance in rural areas—are real, they should not be taken as a fait accompli. Job attributes, including earnings, can be changed in order to make these positions more attractive. Moreover, health worker preferences are heterogeneous, with some choosing to provide health care where it is needed most, and preferences can be moulded, as we discuss later.

**Observation 1: Unbalanced allocation of health workers**

**Table:** 

*Rural areas are intolerable; life is difficult. If you want your child to grow, you cannot accept to work in a rural are. When you go there, you will plunge yourself in a routine, will not be able to increase your living standard, and you eventually become a peasant*. Auxiliary worker in Kigali
*It is Siberia!* Doctor in Kigali
*In a rural area, a doctor kills himself. He looses all elements that make up a real doctor, and will end up with 25% of what he knew*. A doctor in a rural district
*Nurses working in the rural areas are not satisfied and insult the patients; while doctors working in the rural areas become bitter to the point they don’t take hitchhiking patients, and all that because they’re stuck in the rural areas*. User in Kigali
*(…) there’s no plan of development for a doctor working in the rural areas; once you’re affected to the rural areas, it’s as if you’re lost in the rural areas. The lack of this career development for the doctor in a rural area means that once you’re affected or mutated, it’s like a punishment*. Doctor in a rural district

#### Problems related to on-the-job performance

Apart from shortages, health workers’ on-the-job performance forms a second set of problems. In the discussions, health professionals and users identified four categories of challenges. Firstly, many health workers have poor attitudes towards patients and seem to have weak patient management skills. They are frequently impolite and rude, favour some patients above others and often care little about patients’ long waiting times. This is important for users, as 61% of the user quotes are about health worker attitudes and their reception of patients. Absenteeism among health workers is a second problem. Many health workers stay absent from work during parts of the day—sometimes the entire day—and this appears to be mostly related to a second job and is particularly high among health workers in urban areas and among doctors. About 40% of the quotes on absenteeism (26) refer to doctors. A third problem is the presence of corruption and embezzlement among health professionals. Conduct varies from asking bribes to helping patients jump the queue or escape high bills, to asking extra payment for outside-hours services, or falsification of documents. Monetary gifts are at times seen as no more than a token of gratitude. Embezzlement of drugs and materials also occurs, often to be used in the provision of informal care or to be sold on in the market (especially for drugs). The discussions generated 73 quotes on inappropriate behaviour, indicating that it is firmly present. Examples, when used, mostly refer to unorganized and petty corruption. A final problem is the lack of medical skills among health professionals, especially among nurses and auxiliary health workers, who are concerned about their own lack of knowledge, for instance, in how to deal with HIV/AIDS. The latter is confirmed by people living with HIV/AIDS, who complain about the lack of knowledge among health staff.

All four types of performance challenges appear to be far more important in the public than in the private and faith-based sectors. Patients confirm that health worker attitudes are better in private-for-profit and faith-based facilities; health workers point out that absenteeism, corruption and embezzlement are lower in the private sector. Both patients and health workers suggest that this has to do with the higher pay, the superior monitoring and accountability mechanisms and the difference in work culture in the private sector, while health workers in faith-based facilities are also perceived to be more committed. But the respondents suggest that the situation in public facilities is improving with both absenteeism and embezzlement of drugs apparently falling. The implementation of performance-related premiums, the training of health workers on quality assurance and the increased monitoring of health workers by community representatives are perceived as playing an important role.

**Observation 2: Performance problems among health workers**

**Table:** 

**Poor attitudes**
*To receive the patients well, you need sufficient and capable personnel. The faith-based sector has enough personnel and equipment, but the public health centre often lacks personnel and equipment. Therefore people don’t like to go to public health centres*. User in a rural district
*Health workers in the public sector may receive patients badly because they know there are no consequences, but in the private sector the employer is usually around and observes how things are done*. Auxiliary health worker in Kigali
*.. and if you dare saying you feel bad, they react angrily which makes you even sicker*. User in Kigali
*There was a mother who took her ill baby to the hospital asking for drugs and the nurse said ‘Go away, when your baby dies, I will deliver another one for you’; the nurse is still working at the hospital; unfortunately the baby is dead*. User in Kigali
*Health workers [in the public sector] insult patients who are for example dirty*. Auxiliary worker in Kigali
**Absenteeism and shirking**
*Doctors are more absent in the public sector than in the private sector*. Doctor in a rural district
*To some of the remote health centres, you can go and find no one to receive you*. User in a rural district
*When the State has prevented you to take up another job, for example with an NGO. Then, you just act as if you work*. Doctor in Kigali
*Many health workers pretend to work*. Doctor in Kigali
**Corruption and embezzlement**
*Patients paid more than was reported in the register, we’ve found many of those cases*. Auxiliary worker in Kigali
*There may for example be an agreement between the patient and the nurse: the patient leaves at night and in the morning it is observed that the patient left without paying. In fact he has given money to a nurse*. Doctor in Kigali
*Some doctors sign medico-legal documents making things up*. Nurse in Kigali
*The theft of small material is really very frequent but you can never know who did it*. Auxiliary worker in Kigali
*We had a case where nurses embezzled drugs, sold them, took the money of the community and used it for their proper consumption*. Doctor in a rural district
*The embezzlement of drugs had become important but now we require that when leaving the office you show the next person what is not there*. Auxiliary worker in Kigali
**Lack of technical skills**
*There are health centres headed by an auxiliary health worker; this leads to problems regarding the quality of care*. Doctor in Kigali
*In our health centre there’s only one A2 nurse. When she leaves for training, I remain alone and do everything without being properly educated. I do it but it really is a problem.* Auxiliary worker in a rural district
*Our knowledge is not sufficient. The training that is administered does not arrive at our level*. Auxiliary worker in a rural district

### The central role of institutional factors

Four institutional factors emerge from the discussions that have a determining influence on the quality of care provided, by affecting either or both performance or career choice: incentives, monitoring and accountability, norms and workplace culture and intrinsic motivation. We discuss each in turn.

#### Extrinsic incentives

The discussions indicate that insufficient reward is the single most important source of frustration among health workers: 25% of the quotes about low satisfaction refer to low remuneration. Especially lower level health workers indicate that their earnings are not sufficient to satisfy the basic need of family life. Because wages are modest, health workers often engage in secondary activities. This has an effect on their performance, and health workers argue that they would perform better if they would get a higher salary. Other rewards associated with a job also help explain job preferences. Posts in rural areas, for example, come with lower access to infrastructure and good schools, lower availability of secondary activities, and lower access to alternative employment opportunities; they also are also associated with personal and professional isolation, which may limit future career opportunities. Doctors, for instance, have almost no incentive to work in rural areas since they earn more, can specialize, and enjoy a higher quality of life in urban areas. Because of this large difference between urban and rural areas, remoteness benefits, which typically represent only a small percentage of the salary, is likely to have limited effects, although they may help attract some health workers. More detailed quantitative analysis is needed to find out what premium is needed.

Differences in rewards also help explain the choice between sectors, but again, only to some extent. Although many health workers prefer to work for the private for-profit sector, which pays the highest wages, some prefer to work for the public sector because it offers higher job security, access to training, and access to medical care at a reduced cost. Still others prefer to work in the faith-based sector, which follows the public sector salary scales but provides additional benefits, including on the job training. Thus, salaries by themselves seem to have some but limited power to attract health workers to certain positions.

**Observation 3: The importance of incentives for health worker performance and career choice**

**Table:** 

**Earnings**
*Doctors’ salaries should be increased in order that, when he goes to work he does not go to work for just some hours and then goes elsewhere to earn more*. User in Kigali
*If we want a doctor to deliver a good service, he needs to have a good salary, to live, to ensure his family can live, and to ensure that when he treats 10 patients, they are treated well*. Person living with HIV/AIDS
*If our salary were increased, we would work better and, moreover, be extremely happy while at work*. Auxiliary worker in a rural district
*We have friends - lawyers, economists, and others - and when we talk to them, we hear what they earn, and find that they earn much and work little*. Doctor in a rural district
*In the private sector or in an NGO, one earns a lot of money*. Nurse in Kigali
*Public health pays best. They are paid but do not have to work*. Doctor in Kigali
**Other attributes**
*There is the training, medical care, annual salary increase. The most important advantage of the public sector is the access to low cost medical care; we only pay 15% of the bill, even of the drugs*. Nurse in Kigali
**Stability**
*What’s important is the job stability. In the public sector one has more security even if one is not paid as good as in the other sectors*. Nurse in Kigali
*It’s true we’ve been talking about salary and other problems in the public sector, but it’s not like with those Americans [who are investing in public health by starting new NGOs] who are here for a set period only and will leave afterwards, so they can chase you out any time. Then it’s better to earn a small but regular sum of money*. Auxiliary worker in Kigali
*In private health centres, one never signs a contract. You just draw up a convention and when it’s necessary, the employer fires you, but as long as you work well, he keeps you*. Auxiliary worker in Kigali
**Training**
*An advantage of the public sector is that one receives training*. Auxiliary worker in a rural district
*If we had the choice between training and salary increase, we’d choose training*. Nurse in Kigali
*A specialisation is generally obtained in the public sector; it’s far more difficult to specialise in the private sector*. Doctor in Kigali
**Public-private**
*You can’t compare the faith-based sector with the public sector, for example, in certain faith-based hospitals, they have specialists coming over and staying for one or two months and then you receive training*. Doctor in a rural district
Rural urban
*In the public sector, it is encouraged to work in the rural districts and you receive a premium*. Nurse in Kigali
*It’s necessary to have a second job. Experience shows that almost all doctors in Kigali have a second job. In the rural areas, this is not possible because there are no opportunities for a second job*. Doctor in a rural district
*What we would need to be motivated to go to a rural area? A salary increase. Equipment. The possibility for further training. Access to education for the children*. Doctors in a rural district
*Doctors in the rural areas have a particular reputation in the medical corps, they make many mistakes and do things in a mediocre way*. Doctor in Kigali
*In the rural areas, a doctor would kill himself You loose all that makes you a real doctor, you end up with 25% of what you knew*. A doctor in a rural district

#### Monitoring and accountability

Health workers and users have much to say about monitoring and accountability with 80 quotations directly attributed to this theme. They confirm that it is difficult for employers to monitor health worker performance and that monitoring and accountability systems can differ greatly across sectors and locations; they are weaker in the public than in the private and faith-based sectors and less present in remote areas. Users point out that health workers are not sufficiently accountable. Sanctioning for bad performance is unheard of, according to health workers themselves, except for specific and extreme cases of recurrent misbehaviour, like drinking or fighting at the workplace. Especially doctors are weakly supervised. The annual performance evaluation system that is in place in the public sector, which aims at monitoring individual behaviour, is seldom used and perceived as subjective where it is used. The current efforts from the Government of Rwanda to attribute a larger role to community monitoring and to reduce the need for monitoring by making pay more dependent on performance have created high expectations; we evaluate these new interventions in the ‘Discussion’ section.

**Observation 4: Monitoring and accountability**

**Table:** 

*There’s no system of supervision in the public sector*. Doctor in Kigali
*Here in the hospital, a nurse can commit a professional mistake but there is no law that can punish him*. User in Kigali
*In the faith-based sector, health workers are continuously monitored. It’s impossible to have time wasted*. User in a rural district
*In the private sector, you’re sacked even for a minor mistake, but in the public sector you’re sacked only if you’re really impossible*. Auxiliary worker in a rural district
*It also depends on where you work; fraud can occur in public health centres, but in the faith-based or private sector it’s far more difficult since the control is much more rigorous*. Doctor in a rural district
*We have an annual evaluation that should normally be sent to the Ministry of Health, but it is not respected and is not being done regularly*. Nurse in Kigali
*Performance is evaluated using an evaluation form, but it is of no use because there is a problem of objectivity: who evaluates whom and how?*. Nurse in Kigali
*I give everybody “very good”; it’s subjective because there are no objective criteria*. Doctor in Kigali

#### Professional norms and workplace culture

The discussions confirm that professional norms are typically acquired during professional training, and there are indications that the strength of norms varies across schools. Workplace norms are said to vary substantially across sectors. Private for-profit facilities seem to be more client oriented but also more money oriented and have a heavier work load, while health workers in not-for-profit facilities have a reputation for being dedicated. Public sector facilities have the most damaged image and are perceived as having weak work ethics. Some workplace norms have been transformed into bylaws, which health workers are expected to follow. The increasing role of the Ordre des Médecins, a professional self-governing association of medical doctors established in 2001, gives health workers high hopes for monitoring professional norms, enforcing internal rules and providing credible punishment where needed. Nurses have expressed their hopes that they too would have a professional association soon.

**Observation 5: Professional and workplace norms**

**Table:** 

*The regulations are clear, if you are working for the government, you are expected to have a certain attitude*. Doctor in a rural district
*The bylaws consist of a number of rules to follow. If a person does not respect a rule, he is told so and informed about the sanction, the first time orally, the second time in writing. If it happens another time, the sanction is applied*. Nurse in a rural district
*If you compare the public and the faith-based sector, you’ll find that there are more problems in the public sector because the health workers know they cannot be sacked easily*. User in a rural district
*I have the impression that there is a system when it really goes very badly, when it is absolutely clear that there is a problem, then, something is done, the person is blamed or something else*. Doctor in Kigali
*Since the order of doctors has been created there have been investigations undertaken, and some doctors have been suspended*. Doctor in a rural district
*People love to go to the private sector because the health personnel speak kindly. They treat few patients and take time to speak at length with them; consequently, when the patients leave the health centre, they are very happy*. Auxiliary worker in Kigali

#### Intrinsic motivation

Health workers in Rwanda make generous use of a religious vocabulary when describing their motivation to work in the health sector, using words like ‘vocation’, ‘devotion’ and ‘apostolate’. They attribute an important role to motivation, arguing that one needs to have high intrinsic motivation to surmount low pay relative to high effort, poor working conditions, limited career perspectives and a risk of contracting HIV/AIDS. Some argue that intrinsic motivation is related to religious beliefs and that those working for the faith-based sector are ‘more committed’ to their job. In general, the discussions indicate that there is substantial heterogeneity in motivation. And because of its importance, health workers are concerned that the performance initiatives, which provide bonuses according to reaching quantifiable objective, may erode commitment by stimulating a more ‘mechanical’ behaviour.

Since public health workers can to some extent choose the location of their job, those with high intrinsic motivation tend to self-select into positions they like, for instance in the faith-based sector or in rural areas. The for-profit sector, which tends to put higher demands on health workers and provides higher payment, may attract a different profile of health workers. The public sector seems to attract those who plan to continue for further specialization or those who do not find a job in the (for-profit or not-for-profit) private sector.

**Observation 6: Intrinsic motivation**

**Table:** 

*Some say being a health worker is an apostolate*. Doctor in a rural district
*Whether you want it or not: when you study medicine, you have a vocation. If you do not have this vocation, you will fail. A doctor needs to be permanently devoted; a doctor without devotion is not a doctor*. Doctor in Kigali
*When you have a vocation it is impossible to do something else*. Auxiliary worker in a rural district
*There is always this vocation, which pushes you to continue even if conditions are not met: the salary, the equipment, the other colleagues, the professional environment; but since lives are threatened, you are pushed to continue, the situation is more or less comparable in the public, private or faith-based sector*. Doctor in a rural district
*We always work longer than the planned timetable, it’s a vocation; you cannot leave a patient because it’s time to go*. Nurse in Kigali
*Health workers in the faith-based sector are more committed to their work compared to those in the public sector*. Auxiliary worker in a rural district
*Today, health workers only seek money*. Person living with HIV/AIDS

### Institutional innovations to improve performance

Related to the above, three innovations that were in the process of being implemented at the time of the research, received much attention in the discussions: the gradual implementation of performance pay, the establishment of community health committees and the training of health workers in attitudes and patient management skills.

#### Performance pay

In the private for-profit sector, pay is typically related to performance, and workers who perform well receive a higher salary. This has inspired policymakers to introduce performance-related pay in the public sector. In 2001, the Government started the Initiatives for Performance (IP) in a number of public and faith-based facilities, making part of the funding that a facility receives dependent on its performance. Importantly, performance is not measured at the individual level but at the facility level. The reward thus goes to the facility, if it performs well, who determines itself how to distribute the premium among its employees. It may decide to pay higher wages to better performing health workers, or it may also decide to hire extra personnel.

In general, the IP receives a positive evaluation from both health workers and users in our discussions. Health workers monitor each other more than before, and this has improved their performance. IP appears to have had both a quantitative and qualitative effect: it seems to have increased the number of vaccinations, as well as pre- and postnatal care, and curative care (which are all part of the targets); it also seems to have improved the attitudes of health workers towards patients, the general quality of care and the teamwork among health workers. A reported shortage of IP is that while it translates mostly into extra payment—almost half (14 out of 30) of the quotations on performance pay refer to payment modalities—it does not necessarily lead to improved career development. Users’ highlight another potential downside, namely that it encourages health workers to implement certain activities mechanically, thereby eroding the overall quality of care. Thus, although it may induce health workers to put more effort in their job, users fear that effort will be directed towards certain activities, usually the more profitable. Measuring performance at the facility rather than at the individual level reduces this tendency to some extent, by increasing peer monitoring and team motivation. Some health workers are also dissatisfied with the lack of transparency of how the premium is distributed within the facility. Finally, IP has created high expectations but cannot address all shortcomings. Health workers complain that it has not solved the lack of equipment and the shortage of technical skills of health workers.

**Observation 7: Performance pay**

**Table:** 

*A health worker knows that if he does not work well, if he is absent, if he is too late, if his service is not appreciated, this will decrease the premium that the health centre receives. It makes that personnel controls each other. Everybody knows that the one that works badly can be sacked and risks being accused by his colleague; this leads to a higher degree of accountability and higher productivity*. User in Kigali
*You’re the only doctor to consult. But with the IP, one recruits, and instead of one doctor, you’re for example five. So there’s an improvement in the work conditions and you’re no longer stretched*. Doctor in a rural area
*Now, we see that there’s improvement in the motivation of health workers. Regulations have been developed by each health centre and have to be followed. The regulations are such that when they are not followed, part of the premium is withdrawn, which makes the personnel more accountable and more responsible. We are more conscious of what we have to do. The stability of our personnel is also assured by this premium*. Nurse in Kigali
*Patients are better received, the guards are better organised, but as for quality, technically spoken, there has not yet been improvement because there is no technical support, no equipment*. Doctor in Kigali
*In vaccination for example, before the premium we asked ourselves why we had to fetch children that were not vaccinated, but since IP, we are obliged to go and search the one child that has not been vaccinated because we know that this activity is paid for*. Nurse in a rural district
*There’s inequality in the distribution of the premium, even if we do the same tasks and have the same diploma*. Auxiliary worker in Kigali
*It is a good initiative, but performance should not just be acknowledged in financial terms, I think people also want advancement in their career, they want promotion*. Doctor in Kigali
*But what often happens is that after a while, there’s the risk of routine, to carry out lots of activities that are not necessarily of good quality; something that can make the Initiative for Performance perpetual is a constant monitoring system*. Doctor in a rural district

#### Community health committees

As part of a wider move towards decentralization and the involvement of local communities in governance and management of the health centres, in the spirit of the Bamako Initiative, the Government of Rwanda encourages the establishment of Community Health Committees. So while performance pay encourages self-monitoring and sideways monitoring, Community Health Committees increase monitoring from below through community health workers who represent the patient population. One of the risks of this type of decentralization is the capture by local elites [6]. Indeed, those in a privileged position may capture these new powers from the community and use them directly for their own benefit, for example, to employ loyal community members.

The discussions suggest that community health committees help address the lack of accountability of health workers, especially in rural areas. By letting community representatives have a seat on the health committees, they can use their discretionary power to give financial rewards to well-performing health workers and use disciplinary measures to badly behaving health workers. Users argue that the approach has led to an increase in the quality of care, mainly because of lower absenteeism and improved attitudes towards patients. Because information on health worker behaviour is mostly available at the local level, community health workers seem to close the information gap that typically exists between the higher-level health administrators and local health service providers. The fact that Rwanda has a high population density, also in rural areas, may be an important contributing factor to the success of the approach.

**Observation 8: Community monitoring**

**Table:** 

*The health committees give these performance bonuses; if someone is absent, the bonus is stopped. Donors also give bonuses; if someone comes too late three times in a month, the bonus is stopped*. Auxiliary worker in Kigali
*To find a health worker reading a book when you visit a health centre does not happen anymore. It happened before, but now, they fear the health community workers*. User in a rural district
*When a nurse doesn’t interact in the right way with patients, community health counsellors can take action; they can decide to fire him*. User in Kigali
*Since the decentralisation the health centre is managed by more than one person, and there is a representative of the State, but there is also a representative of the population, who sits on the health committee. This committee can decide, for example, to pay the rent of a health worker, or to give him a premium, etc.. All this is on top of his salary. Sometimes, workers with an equal qualification do not receive the same bonus*. Auxiliary worker in Kigali
*The health committees give performance bonuses; if someone is absent, for instance, thebonus is stopped. Donors also give bonuses; if someone comes too late three times in a month, the bonus is stopped*. Auxiliary worker in Kigali
*Since community health assistants control the functioning of the health centre, these problems do not exist anymore. If a nurse does not behave well towards patients, community health assistants take decisions for this nurse. They have the power to hire and fire. This is why the health centres function better today*. User in Kigali
*Today, representatives of the population control the functioning of the health centre; if you’re absent, even an ordinary citizen can accuse you by saying for example ‘I’ve seen him in that place and he wasn’t working’. When the population has a meeting, the one who has seen you or has had a problem will make it public*. Auxiliary worker in Kigali
*Sometimes the population nominally accuses personnel of the public hospital in the newspapers*. Auxiliary worker in Kigali
*If the health committee is willing, if you’ve worked well, they can take money out of the financial reserves of the health centre and thank you. For example, at the end of each year, I don’t know whether it’s to thank us, they take us where we want, we eat, we drink and the health committee pays the bill*. Auxiliary worker in Kigali

#### Training

The government of Rwanda has started to increase the provision of training for health professionals to change their attitudes and practices. The discussions suggest that this training has been effective. Health workers argue that it has helped to improve the quality of care. Users draw no causal conclusion, but it is hard to say whether this is because the initiative is too recent, because its effects are limited or because users do not know about the training. Future evaluations may help shed light on this.

**Observation 9: Training**

**Table:** 

*Since the training on quality assurance there’s improvement in the attitude of health workers*. Auxiliary worker in Kigali
*In the past, before the training on HIV, someone who was suspected to be HIV positive was abandoned to himself, he was not treated, but that actually does not exist anymore, with the training, we help them, we treat them*. Nurse in a rural district

## Revisiting the findings in 2015

A number of studies and reports that have taken place since 2005 document whether and how things have changed. Three observations come to the fore. First, despite improvements, substantial problems and challenges remain, similar to those identified in the 2005 group discussions. Second, interventions were implemented have had mixed impact. Third, institutional factors remain a central force that helps explain failures and successes of health care delivery. We discuss each in turn.

### Despite improvement, problems and challenges remain

There have been considerable improvements in health outcomes in Rwanda since 2005. Maternal mortality rate fell by 72% (to 210 deaths per 100 000 live births in 2015), under 5 mortality decreased considerably (from 152 to 50 per 1000 live births), while life expectancy increased to 66 years [71]. Attribution of these changes to specific programmes remains a challenge, as discussed below. And despite these improvements, substantial problems and challenges remain, both in terms of outcomes—for example, the sustained chronic malnutrition (38%) and neonatal death (39%) [71]—and in terms of processes and inputs. Rwanda remains ranked in the bottom 20% for human development, poverty and several health indicators [74]. In what follows, we assess the extent to which this is related to poor personnel performance, using the two dimensions identified in the group discussions: the number and distribution of health workers, and health worker on the job performance.

Despite considerable improvements in health worker numbers, including community health workers, substantial shortages remain. Recent figures suggest an overall health worker density of 0.72 per 1000 inhabitants, with the number of doctors having increased from 0.002 to 0.06 doctors per 1000 population, which is still far below the WHO recommended 2.5 health worker per 1000 inhabitants [65, 99]. More detailed figures suggest a gap of 50% between available and required staff, varying from 34% for General Practitioners to 86% for midwives [66, 67]. In-depth quantitative analyses highlight the remaining skewed distribution of doctors and nurses in favour of urban areas [85] and summarizes the key challenges as threefold: a general shortage of midwives; a maldistribution of doctors across hospitals, with shortages in some hospitals but not others; and a shortage of nurses’ time spent on health care rather than receiving and counselling patients, which should be done by social workers; the latter are overrepresented in hospitals (159% of what is needed) and lacking in health centres (20% of what is needed), [64].

Other studies detail the challenges with health workers’ on-the-job performance. Lannes [50], using a simple index per facility, finds that average health workforce productivity went down over the considered period.^8^ One third of health workers were reported absent in primary health care facilities, and this increased further with an estimated 16% 1 year later. It is poorly understood why this trend has occurred.

Another study assesses health workers’ on-the-job performance and found that less than 10% of patients were routinely screened for chronic conditions like anaemia, malnutrition, HIV or tuberculosis [100].^9^ Nurses were found to correctly diagnose 50% of patient complaints and to provide the correct treatment 44% of the time. The median duration of consultation was 6 min. Prevention counselling had low rates. The quality of decision-making was independent of the nurse’s experience. The study concluded every aspect of the routine to be in need of strengthening and quality improvement and identified needs of nurse training and standardization of care. Brown and McSharry [20] illustrate the wanting accuracy of child growth measurement and find that use of electronic tools would allow for superior tracking of health information in rural and urban Rwanda.

These substantial shortcomings in performance confirm those observed in other studies. A cross-country ranking of health care delivery across Rwanda, Tanzania and Uganda indicated that Rwanda performed only slightly better than its neighbours, despite scoring lower on some health outcomes, confirming structural weaknesses [1]. Detailed studies on these two other countries provide a depressing picture of a human resources characterized by high absenteeism (55 and 21% on average),^10^ limited time on the job, high variability in case load,^11^ and low accuracy in diagnosis, with between 19 and 57% providing a correct diagnosis, depending on the condition^12^ ([107], 2017).

Policymakers have become increasingly aware of these challenges, and improving Human Resources for Health has now become a central part of the Ministry of Health’s strategy, as expressed in their current HRH Strategic Plan 2015–2018 and HRH Operational plan 2016–2018 [65, 67].

### Mixed impact of interventions and innovations

The lower than expected performance documented above relates to the mixed impact of four policy interventions that have been implemented since 2005: increases in health worker numbers and skills, performance-based pay, changes in governance of the health sector and the rollout of mutual health insurance. All of these changes were initiated before 2005, as is also clear from the discussions, which frequently refer to them, and have been scaled up and accelerated since then. Overall, we find that these innovations had mixed effects, bringing about some improvements but also unintended consequences; if anything, they seem to confirm the key findings from the qualitative research and underline the primary role of institutional factors. We discuss each of them in turn.

A second change that took place is the further rollout of the Initiatives of Performance (IP). After initial years, a pilot study was implemented in 2005 to test the pay for performance programme, which was then scaled up over the subsequent years.^13^ Much speculation took place about the programme’s impact (positive or negative), but evidence remained absent until more recently. This more recent rigorous evaluation suggests heterogeneous impact, with beneficial effects on the use and quality of child and maternal care and on the quality of prenatal care [7]. Impact was largest on those services with the highest payment rates and requiring the least effort from the service provider.^14^ These findings suggest that IP does affect some dimensions of the average health worker’s behaviour, but not others, and remains agnostic about health worker individual responses.

Later studies look at other impacts of IP. They find that absenteeism reduced in IP facilities, but only by a modest 6% [50]. IP had a negative effect on recruitment: less nurses were recruited in facilities that have P4P, and this led to lower replacement, resulting in 21% fewer nurses in treatment facilities compared to control facilities [50]. The reason for this remains unclear. It may be that performance pay facilities are more efficient in service delivery and thus require fewer staff to perform the work or that the IP workplace is less attractive for prospective health workers. IP also had a positive impact on overall patient satisfaction and satisfaction with waiting times, but not satisfaction with provider or cleanliness ([50]). Extension of the previous evaluation finds that IP primarily increased access for patients who are relatively easier to reach and who also tend to be the more affluent, but had less impact on the poorest members of society [51].The impact of pay for performance on community health workers is the subject of another recent large scale study, which finds no effect on the coverage of services that were targeted or on the behaviour and attitudes of the community health workers who were incentivized [90].

Modifications in governance of the health sector constitute a third area of intervention. Reforms aimed at further encouraging and formalizing the establishment of Community Health Committees increased decentralization. In 2008, health facilities were granted formal financial and administrative autonomy. Unfortunately, there is no formal evaluation of the different steps of decentralization, including the increased role of communities in the management of facilities. One report, relying on descriptive data, suggests decentralization had a positive impact on health governance mostly related to accountability, responsiveness, efficiency and effectiveness [97], but attribution and causal inference remain a challenge with the descriptive data. It also remains unclear what the overall impact on health worker behaviour is, as increased monitoring often invites strategic behaviour and ‘gaming’ [46] and opens opportunities for local capture.

A final policy intervention has been the further scale-up of Mutual Health Insurance.^15^ Between 2000 and 2003, building on previous experience, over 100 Mutual Health Insurance schemes were implemented [81]. This led to an estimated 27% of the population being covered with Mutual Health Insurance in 2004, rising to 74% having some form of health insurance cover in 2007 [69]. An evaluation study in 2011 highlights the scheme’s association with demand-side changes, including increases in utilization and increased protection of users against sudden health expenditures. While this may have contributed to improvements in health outcomes, there has been no study on the impact on health worker performance and quality of care. Anecdotal evidence suggests that it has put more strain on health personnel: outreach may have gone up, but it is unclear what the impact on quality of care has been.

### A central role of micropersonnel institutions

The studies consulted in phase two confirm a key role for the four institutional factors identified in the first phase of the research. The role of earnings as incentives is generally recognized in the studies, including those on performance pay. One study also demonstrates the role of earnings for job choice and finds that close to 40% of nursing students and 7% of doctor students are willing to work in a rural area at the going wages, while an average premium of 71 and 57%, respectively, would be required to get 80% of new nurses and doctors to take up a post in a rural area (where 80% of the population lives) [85]. The role of other job attributes, including access to training, general career support and possibilities for career planning, is mentioned in several studies. Overall, the picture that emerges is one of health workers receiving improved but still mixed incentives, and limited support, to perform well.

The second set of factors, monitoring and accountability, also receive considerable attention in the studies and reports. Nurses and community health workers in particular seem to receive limited supervision and monitoring [26, 101]. Interventions that aim at increasing health worker responsiveness and accountability, like IP and community monitoring, seem to have had some but mixed success.

Professional norms represent a common theme throughout the above studies, which underline their importance for performance benchmarks for health worker of all levels [50, 90, 101]. The role of motivation, while long recognized, is also receiving increased attention in the recent analysis. Studies on community health workers highlight the central role of motivation for their performance, while others analyse its importance for health worker job choice (see [26, 90, 85]).

## Discussion

The problems identified in the group discussions and confirmed in later studies are consistent with those arising in other contexts and countries, in sub-Sahara Africa and beyond. Qualitative research for Ethiopia identifies very similar challenges [61], while work by Jaffre and Olivier de Sardan [45] find comparable problems for most capital cities in Francophone West Africa. Recent work in this journal and elsewhere also identifies similar issues (see for instance [13, 37, 58]).

The findings underline, first of all, that health workers make conscious, albeit constrained, choices about where, when and how to work. While this may seem obvious, it is in contrast with much of the work on human resources in the health sector, which still views health workers as passive actors and assumes that they have uniform preferences. The findings also indicate that these choices depend crucially on four institutional factors: extrinsic incentives, monitoring arrangements, norms and intrinsic motivation. All four factors affect both the career choice and performance of health workers. In what follows, we discuss each of these four factors in turn.

### Extrinsic incentives

Health workers see remuneration and other pecuniary and nonpecuniary benefits as a central issue when talking about performance. This fits with the classic view of management and economics that people who are paid more, perform better. There is, however, little consensus why this is the case, as the relationship can go both ways. Workers may perform better because they are paid more, or they may receive higher payment because they perform better. In a public sector context, where rewards are heavily regulated, the question then becomes whether the rewards are sufficiently high both to make existing health workers perform well and to attract, and retain, high-performing candidates.

Empirical evidence on the relationship between payment and performance in the health sector remains limited. Some insights can be gained from studies on the effect of wages on labour supply. Evidence for high-income countries finds estimated elasticities of labour supply with respect to wages to be low,^16^ but the scarce evidence for developing countries suggests a higher responsiveness of health workers to wages in this context. One study finds a short-run elasticity of labour supply with respect to wages of 0.58 for self-employed practitioners in China [77]. Recent work on absenteeism among health workers confirms that there are limits to the power of incentives, with often more than one third of health workers being absent, whatever their salary.^17^ The strongest correlates of absenteeism seem to be non-wage factors, including facility conditions, provision of housing and cost of getting to work. A separate set of studies looks at the impact of earnings on corrupt behaviour on the job and suggests that its effects are small. Barr et al. [8], for example, using a behavioural experiment, find that earnings reduce embezzlement but that the effect is limited. Van Rijckeghem and Weder [103], using cross-country data, find that higher public servant salaries (relative to manufacturing wages) are associated with less corruption, but neither Treisman [95] nor Rauch and Evans [78] have been able to replicate this result. They do find suggestive evidence, however, that employment security and recruitment and promotion have a more important effect on corruption, suggesting that job attributes in a more general sense have a larger effect on performance.

Besides their impact on labour supply and on-the-job performance, incentives and rewards also affect health workers’ career choices. Compensating wage differential theory argues that jobs with less attractive attributes typically offer higher wages. A number of studies show that willingness to work in a rural area is strongly influenced by pay^18^ and other benefits, as also mentioned in the discussions.^19^ This is confirmed by quantitative research with future health workers in their final year of study [85]. Remuneration can also differ across sectors, more specifically the public, the private for-profit, and the not-for profit sectors. Results for other countries, including Ethiopia and Ghana, indicate that many health workers prefer to work in the private for-profit sector, which typically offers higher salaries and better physical infrastructure [53, 89].

The discussions suggest that access to training is an important factor for job choice. This is in line with studies for other countries,^20^ and often seems to be an important motivation to work in the public sector, which tends to provide the most opportunities for further training and specialization. However, senior management may not select or allocate training opportunities based on professional need or as part of staff career development. Rural jobs may provide lower access to training, but they can offer thorough first-job experience, which is strongly appreciated by some health workers and employers alike.

The findings of our research confirm that incentives and rewards matter but that health workers take a much broader set of incentives into account than the ones that are usually considered. Apart from earnings, which include the health worker’s salary and her compensation for overtime, other financial benefits like the health worker’s pension, the per diem she receives for fieldwork or training and the reimbursement of health care costs for his family members are also important. Non-financial benefits, like access to training, job security, chances of promotion and other contractual stipulations that affect career planning, are also recognized as important job attributes. Finally, indirect factors that are associated with the place of work, like access to quality housing and infrastructure, including good education for their children, and access to other income opportunities, including for instance a second job, also play an important role (see [87]).

Summarized, incentives in this more general sense seem to be important to help explain health workers’ career choice and performance, probably more so than existing empirical research—which typically focuses on earnings in narrow sense—indicates.

### Monitoring and accountability

Monitoring and accountability form a second factor that affect both the performance and career choice of health professionals. This fits with theoretical considerations which emphasize that, when work is characterized by multiple tasks for which the outcomes are difficult to observe, employment contracts are highly incomplete leaving plenty of scope for moral hazard.^21^ The role of monitoring has attracted growing attention in the analysis of service delivery and public service. Generally speaking, monitoring can take different forms: agents can be monitored from above—in our case by their superior or a representative (an inspector); from below—by their clients or the community they serve; or from aside—by their colleagues and peers. Regarding the subject of monitoring, there are two approaches: monitoring can focus on outcomes or on processes. An important question is how much monitoring is needed. Too little monitoring is ineffective, while too much is inefficient and may negatively affect performance as it may erode motivation. Also important is the sanctioning mechanism. Monitoring is likely to have limited effect without credible sanctioning. Serneels et al. [86], studying absenteeism in the health sector in Ethiopia and Rwanda, observe that punishment in case of detection is very unlikely, rendering any monitoring strategy mostly ineffective. There is, however, limited detailed empirical evidence on the role of monitoring and accountability because one needs variation in monitoring regimes to study its impact. Bjorkman and Svenson carry out a fascinating investigation of the potential benefits from community monitoring, which will be discussed in the ‘Community health committees’ section. Existing work on the education sector indicates that monitoring can have large effects.^22^ But supervision is not without challenges. Health workers ‘gaming’ the system has been a common concern, especially when stakes attached to the reported behaviour are high.^23^ One example for Udaipur shows how the initial success of a scale-up of automated monitoring of health worker attendance in the public sector was eroded in the medium term, because health workers had increased their ‘exempt’ days, a consequence of induced machine problems. Evidence from behavioural experiments also suggests that how a monitor is appointed—for example, whether he is elected by the community or not—can also have important effects (see [8]). Concerns of ‘gaming’ are highly relevant for the health sector in Rwanda, where increased monitoring by use of a database of indicators [21] combined with performance pay may steer health worker behaviour in the direction of improving indicators, possibly eroding the overall quality of services [46]. It may also induce falsifying data to obtain more financial means.^24^

Summarized, the findings indicate that the arrangements regarding monitoring and accountability often tend to be weak. Sanctioning, for example, is usually absent or lacks credibility. However, given the wide range of tasks in which a health worker is typically involved, while the need for monitoring is high, the very nature of the work makes monitoring a challenge, calling for more innovative approaches. Performance pay and community monitoring seem to be effective in some ways, but a concern remains about keeping the consequences of gaming the system to a minimum. Recent work suggest that training, coupled with regular local follow-up supervision, may be a promising route [101].

### Norms

The group discussions highlight the importance of norms related to professional and workplace culture. Professional norms are typically acquired during medical education and training, and the study suggests that the strength of these norms may vary across schools. Later research confirms the importance of such performance benchmarks, standardized practice and norms [101].

Norms can generally be seen as an implicit way of monitoring or an implicit contract enforcement device [35]. Professional norms are believed to be fundamental in constraining opportunistic behaviour among service providers, especially in the health sector (see for example Frank [40, 36, 37, 93]. For our purpose, we see three relevant levels of norms: society-wide norms, professional (peer group) norms and workplace norms. Society-wide norms are relevant because one expects that, ceteris paribus, corruption among health workers will be higher if there is more corruption in society at large. Peer group norms are important since they determine professional success and recognition; they are guarded by a professional body, like the Order of Physicians. At the level of the workplace, norms provide a way of monitoring daily behaviour like attitudes towards patients, work ethic and engagement in corrupt activities.

The discussions suggest that health workers who are surrounded by a culture of poor performance; for example, those in the public sector are more likely to also perform poorly, while those working in a place encouraging performance—for example in the faith-based sector—perform much better while receiving the same salary. That norms directly affect a worker’s performance and career choice confirms both existing theory and evidence, suggesting that workers internalize norms over time—be it at the level of the workplace culture, the profession or society at large—to guide their own behaviour [30].^25^ Vasan (2015), in a pilot study, offers a detailed example for Rwanda, demonstrating how training that aims at improving health workers’ adherence to preferred practice can have significant effects on health worker behaviour. Recent work for high- and middle-income countries suggests that management practices matter for clinical outcomes of hospitals [17]. Little rigorous evidence exist on the role of workplace culture and management in low-income countries and beyond hospitals. One study illustrates its potential importance, observing that absenteeism among health workers in Rwanda is higher in facilities without a head of facility present ([50], p. 84). While this is a topic of great interest, more research is needed.

### Intrinsic motivation

In the group discussions, health workers as well as patients talk about the importance of ‘vocation’ and how ‘commitment’ may be different across sectors. This suggested role of intrinsic motivation for both performance and career choice has historically been recognized for the health sector (see [30]). Recent empirical studies for Rwanda confirm its role [26, 85, 90],

Intrinsic motivation has received increased theoretical attention.^26^ It is typically defined as the urge to do something for its own sake and comes about as the internalization of norms over time. Since this is a slow process, intrinsic motivation is considered exogenous in the short run. It affects output, in the sense that more motivated health workers tend to perform better. It can be seen as a self-enforcing contract that leads to a higher performance outcome [30].^27^ A weakness of many existing payment schemes is that they do not take into account the heterogeneity in motivation, an issue that performance pay may be able to help address to some extent.

Intrinsic motivation also affects career choice as it is highly correlated with career preference [87]. Individuals prefer a job that satisfies their intrinsic motivation. An important question is therefore how candidates are allocated to jobs. In Rwanda, matching takes place through a largely unregulated market—thus taking into account differences in preferences and intrinsic motivation. Recent evidence for Zambia demonstrates that the type of motivation of health workers matters, showing that newly recruited community health workers, who aspire to becoming a ‘highly trained health worker’, perform better than those who primarily want to ‘learn about health issues in the community’ [4]. Combined survey and lab-experimental evidence for Rwanda indicates that health workers with higher pro-social motivations choose to work in not-for-profit organizations [88].

Although intrinsic motivation is considered constant in the short term, it may change in the long term for two reasons. First, new norms may be internalized over time. For instance, health workers who happen to work in a corrupt environment may revise their norms and get socialized into corruption (see [8]). Second, extrinsic incentives may crowd out intrinsic motivation. Seabright [82] illustrates how satisfaction derived from intrinsic motivation may conflict with that derived from extrinsic incentives, explaining why paying for activities that are done out of intrinsic motivation may reduce performance. Titmuss [94] makes a similar point much earlier emphasizing the importance of altruism in the organization of blood donation. Existing evidence is inconclusive whether such crowding out takes place and how important it is. A recent randomized experiment on mission matching among community health workers in Zambia suggests that incentives can also crowd in motivation and improve performance as well as job choice (see [3], [83], respectively). These results call for more analysis of how and where intrinsic motivation matters.

### Institutional innovations

Three institutional innovations were highlighted during the discussions and in later studies as most relevant for personnel performance: the implementation of performance pay, the increased role of community health committees and enhanced provision of professional training.

#### Performance pay

Health workers as well as patients brought up a range of issues related to performance pay. Later studies carried out formal evaluations of different aspects of IP. One key study finds that IP led to an increase in use of service with highest payment and lowest effort, accompanied by a possible increase in quality of care [7], with larger effects for relatively better off patients. Follow-up studies find that it positively affected process quality, but not structural quality [72], possibly increased work force productivity [50], reduced absenteeism [50, 72], had negative effects on recruitment and positive impact on some aspects of patient satisfaction (waiting times), but not others (cleanliness) [50]. Where it was successful, this is believed to have relied crucially on performance being measured and incentives being offered at the facility level. This is important for a number of reasons. The multidimensional nature of health makes it difficult to summarize health outcomes in one compact indicator. Moreover, with some dimensions measured more easily than others, such a measure would steer performance in a specific direction. The complementarity of tasks carried out by different health workers is another argument in favour of rewarding performance at the facility level. This approach also provides an opportunity to strengthen facility management where rewards linked to individual performance may weaken it.

These results are mirrored by a recent systematic review of eight studies in five developing countries,^28^ which confirms that performance pay can have positive effects on maternal and neonatal health outcomes, but has possibly negative effects on structural quality [27].

On the whole, the existing evidence suggests that result-based finance increases the volume of services delivered with some evidence that it also improves the quality of care [105]. A recent study for Cambodia shows that the number of births occurring in public health facilities increased, but the effects also depended on household wealth [102]. Evidence on absenteeism and availability of health care providers is consistent with the findings from other studies on result-based finance [44, 46]; evidence on the impact on productivity is scarce and inconclusive. The Rwanda results on recruitment contrast with findings for DRC where performance pay led to recruitment of more staff [92]. Other recent overview studies, focusing mostly on high-income countries, suggest a variety of possible effects,^29^ underlining the need for more systematic and comparative analysis.

#### Community health committees

The possible beneficial role of community health committees has been widely discussed in the literature in descriptive terms. Much less is known whether this institutional change had a causal impact on the behaviour of health workers and the health outcomes of patients.^30^ A study in Uganda provides a welcome exception and finds that activating community involvement can have substantial effects [15]. Providing an intensive intervention where information is provided to both community members and staff, and both parties are activated in different meetings and then brought together, the study finds large impacts, including substantial increases in utilization, improved health, reduced child mortality and increased child weight. A follow-up study compares the effect of this intervention combining provision of information and activation of staff and community, with the impact of an activation intervention only, where no additional information is provided. In contrast to the former, the latter is found to have little impact on health worker performance and the quantity and quality of health care. The study concludes that enhanced participation alone has little impact without providing additional information. Given its design, it cannot say much about the contribution of participation.

Other studies find more limited impact of increased community involvement. Phillips et al. [76], using a randomized evaluation of community-based provision of nutrition information to mothers of young children, find that including volunteers in health service delivery in Ghana did change health practices but had no effect on health outcomes like child growth, but this changed when they worked alongside trained nurses. Similarly, a study in Nepal finds that the involvement of local community facilitators improved health behaviours, but the effect on outcomes was less clear cut [55]. Tripathy et al. [96] find that local facilitators reduced neonatal deaths and increased breastfeeding at 6 weeks but observed little difference in health-seeking behaviour in Orissa and Jharkland.

#### Training

The discussions suggested training as a way forward, and this was confirmed by later studies which emphasized the potential impact of improved skills [21]. Enhanced training of health workers is often seen as an important way forward to ameliorating the quality of health care. There are clear examples where the curriculum does not capture local disease realities, particularly the disease burden of the poor and those in rural areas, leaving ample room for improvement. Mozambique (non-physician) surgeons, for instance, until recently received no training in HIV/AIDS, even though it was the most common disease they treated. Over the last two decades, many countries have updated their curriculums, with the Malawi approach that tailors content of the training to meeting the community’s most pressing needs, often used as a model. Recent examples are South Africa’s Walter Sisulu University, community-based programmes at Jimma University in Ethiopia and University of Gezira in Sudan, as well as initiatives at Makerere University in Uganda, as well as the National University of Rwanda, who made epidemiology-based curriculum revisions. However, the scarce existing evidence indicates that, on the whole, health workers know what to do; the failure lies in applying their knowledge [28]. This suggests that the traditional focus on training, emphasizing the accumulation of knowledge skills, should be extended to building non-cognitive, socio-emotional skills, in order to ameliorate attitudes and shift norms, such that existing knowledge is applied.

The earlier mentioned pilot study by Vasan (2015) illustrates how a training that aims at improving health workers’ adherence to preferred practice has significant effects on health worker behaviour. A recent twinning programme between Rwanda and US professionals shows potential promise [99].

These examples highlight that, while health worker norms are often seen as exogenous and difficult to change, training may offer a potential way to shift professional norms. Teaching health workers how to increase the quality of care and improve their patient management skills can increase awareness and improve attitudes, whether the training is general or focuses on specific groups of patients, like people living with HIV/AIDS. Training may also have long-term effects, as norms tend to be internalized over time, potentially shifting motivation more permanently. Recent work suggests that motivation among health workers can be fostered. Further work is needed in this largely unexplored area [19].

### Ways forward

So how does this inform future research and policy reform? What is the appropriate theoretical framework, what type of research is needed and what are feasible hypotheses? Our findings indicate that microeconomic and personnel institutional factors play a key role in explaining both health workers’ on-the-job performance and career choice, two outcomes that are best analysed separately. To understand health worker performance, the classic principal agent model provides a good starting point but needs to be expanded beyond incentives and monitoring to also take into account intrinsic motivation and norms. To analyse health worker career choice, standard models from labour economics provide a good starting point but again need to be extended to allow for a role of workplace culture and health worker motivation.

A next step is to test with quantitative research the causal hypothesis generated by this and other qualitative research. Rigorous evidence that tests the causal effect of the four institutional factors on health worker performance and career choice is growing but still thin. A commonly raised objection is that there is (too) little observed variation in these factors within a specific context. The results of our study indicates that this is not necessarily the case as incentive design, monitoring arrangements, workplace culture and the motivation of health workers can vary considerably across sectors, between urban and rural areas, and over time. This opens up important opportunities. A handful of recent efforts makes creative use of randomized control trials to do just that, testing the causal impacts of performance pay [7], community monitoring [15] and intrinsic motivation [3]. Another emerging strand of work makes use of behavioural games to gain further insights on mechanisms and pathways, either as standalone lab-in-the field games (see [8]) or as games combined with RCTs (see [10]). The implementation of and interest in fresh initiatives—like performance pay, the establishment of community health committees and improved training—in the case of Rwanda, can create further opportunities to improve our understanding of how these institutional factors can help improve health worker performance and career choice and ultimately ameliorate health outcomes.

Rwanda’s Ministry of Health’s current Human Resources for Health (HRH) Strategic Plan and Operational Plan have adopted many of the issues identified in this study. Focusing on how to fill the gaps in numbers and performance of human resources, the strategic plan sets out an ambitious agenda with six strategic HRH objectives: (i) strengthen the collegial approach; (ii) increase quantity, scope and quality; (iii) strengthen the capacity of teaching institutions; (iv) improve the capacity to attract, recruit and retain and motivate health professionals; (v) enhance the financing of HRH, including innovative approaches; and (vi) improve monitoring and evaluation of HRH [66, 67]. These are then translated in interventions and in implementing activities as part of the HRH Operational plan.^31^ The planned activities speak directly to the four factors raised in this study: remuneration, monitoring and accountability, professional and workplace norms and motivation and professional training, illustrating the operational potential of this framework.

## Conclusion

Personnel institutions are key for development, but our understanding of their role for health service delivery is still shallow. With the growing consensus that health worker performance is at the core of health care, the question arises how existing institutional design affects health worker behaviour and how it can contribute to improvement in health outcomes. Using qualitative semi-structured research, we held discussions with health workers and users of health services in Rwanda, a country with common health care problems and a dynamic institutional environment. The objective of this research was to identify the problems and challenges with health worker behaviour and to explore key microeconomic and personnel institutional factors that help to explain this behaviour. We then revisit the results of this study 10 years later to evaluate the findings and observe that, despite a number of interventions, the findings remain highly relevant. This is also reflected in the Ministry’s current strategic and operational plans, which aim to address the issues raised in this study.

The challenges we identify are similar to those observed in other countries in Sub-Sahara Africa and beyond and include issues both related to the size and distribution of the workforce, as well as to health workers’ on-the-job performance. The former include a shortage of health workers for specific posts like those in rural areas, in the public sector and in high HIV/AIDS-infected areas. Performance problems, identified by both health workers and patients, include the poor attitudes of health workers towards patients, their frequent absenteeism, their engagement in corruption and embezzlement and their lack of medical and patient management skills, confirming the picture painted by similar research in other countries.

Regarding explanatory factors, four institutional factors that affect health worker behaviour and allocation emerge: extrinsic incentives, monitoring, norms and intrinsic motivation. On extrinsic incentives, our findings underline the need to consider a broader range of incentives, beyond the narrow focus of earnings. The findings also underline the importance of monitoring, or the lack thereof, as well as the key role that norms and intrinsic motivation play. Both the initial interviews and later studies identify innovative initiatives that show promise but also need close evaluation to address their shortcomings; these include performance pay, community monitoring and improved training of health workers. Future study and evaluation is needed to help fine tune these approaches.

## Footnotes

1 See for example Jaffre and Olivier de Sardan [45] for urban areas in Francophone Africa, McPake et al. [59] on coping strategies, Ferrinho et al. [36] on dual practice, Belli et al. [12] on informal charging and corruption, Franco et al. [39] on motivation and Lindelow and Serneels [54] on performance problems of health workers in general in Ethiopia. Early quantitative evidence on absenteeism (see [22]) has been confirmed and extended, documenting poor performance in other dimensions, by recent work for Kenya, Mozambique, Senegal, Tanzania, Togo and Uganda and under way for other African countries (see http://www.sdindicators.org//). These studies also show weaknesses in terms of case load, diagnostic accuracy and adherence to clinical guidelines, as well as challenges with the availability of drugs and quality of infrastructure.

2 North’s [73] classic definition of institutions refers to rules, incentive structures and organisations and is well suited for the issues that come up in this study. Emerging work on personnel economics of the state underline the promise of this approach (see for instance [38]).

3 Note that institutions can be seen as building blocks of systems, which focus on the linkages between these building blocks ([43]).

4 It had less than one fourth physicians per 1000 inhabitants compared to Sub-Sahara Africa as a whole, less than one half nurses and midwives, less that a sixteenth dentists and technicians and about half the pharmacists and technicians, in 2005 [106].

5 A key strength of group interviews lies in the interaction between group members. Group discussions—in contrast to individual interviews—allow researchers to elicit a multitude of views and contrast these. Group discussions may also function as an ‘information quality filter’: highly individual or extreme points of view will provoke disagreement from other participants. Pragmatically, they permit the collection of wide data in a relatively short time span.

6 Selection criteria were well respected overall. Some difficulties arose in finding rural female doctors and health workers active in rural private facilities. Equality of participants in each group is important to enable comparison between groups. Since the group with PLWHA will be considered on its own, nine participants were allowed.

7 Each script focused on a number of issues to which a prompt or trigger question is associated. Five different scripts were developed and can be obtained upon simple request. At the start of the group discussions, participants were informed about the objective of the study, its independent academic character warranted full confidentiality, and were invited to sign a consent form. At the end of the discussion, participants filled out an information sheet and were reimbursed for transport costs.

8 The service index is a weighted sum of the volume of the main health services produced in the primary health care facilities. This downward trend has to be interpreted with caution as it cannot say anything about quality. A high indicator may coincide with low quality of care.

9 The study focuses on adult and adolescents primary care delivery in eight rural health centres in southeast Rwanda and observed close to 500 consultations.

10 Carrying out surprise visits, the studies find that 45% of health providers were absent in Uganda, with the highest absence rate (55% absence) among nurses in rural areas. In Tanzania, 21% was absent. In Uganda, the majority of these were approved absence, and the study concludes that these ‘are thus within management’s power to influence suggesting that the problem of low provider effort is largely a reflection of suboptimal management of human resources.’ An earlier pilot study found time spent counselling patients per clinician per day to be 36 (26) minutes in urban (rural) Tanzania.

11 Case load is often seen as a potential indicator of the consequences of health worker shortages, which may cause a rise in caseload and compromise service quality.

12 Focusing on specific conditions, health workers were found to provide a correct diagnosis for 58% of the tracer conditions in Uganda and 57% in Tanzania (for instance, diarrhoea with dehydration and malaria with anaemia). In just over a third of the cases (35%) did they provide the correct clinical guidelines for these conditions in either country. For maternal and neonatal complications, only 19% followed the correct treatment actions in Uganda.

13 The project’s budget rose from USD 0.8 million in 2004 to USD 8.9 million in 2007 [60].

14 Rusa et al. [80] evaluating an early pilot version of the intervention—which was strongly adapted before scale-up—argue that improvements in utilization during the pilot were primarily in areas that had not previously been well organised.

15 While the scheme goes under the name of ‘Community based health insurance’, it does not have the typical characteristics ascribed to these types of schemes. It is strongly managed from above, owned by the state and seems to function more like a parastatal, with compulsory membership through payment of a flat fee. Some have argued that the scheme acts more like a tax.

16 Two overview studies indicate that the responsiveness of labour supply of nurses to a rise in wages may be limited in high-income countries, around 0.30 [91] or often close to zero [2].

17 Chaudhury et al. [22] were the first to provide systematic evidence of health worker absenteeism across countries using unannounced visits to primary health clinics in Bangladesh, Ecuador, India, Indonesia, Peru, and Uganda. They found that more than a third of health workers were absent. A study on India reports close to 40% absenteeism rates, ranging from 30% in Madhya Pradesh to over 67% in Bihar. An earlier study in rural Udaipur District in Rajasthan, measures absence more intensively through weekly unannounced visits on random days for one year, and found average absence rate of 36%, and 45% in rural subcenters [5]. Similar figures are found in a more recent survey of health centres in Rajasthan, India (27% in general and 36% among doctors, see [23]). In Sub-Saharan Africa. Bjorkman and Svensson [15] observe an absence rate of 47% in Uganda. Another study finds a similar figure (48%) in Bushenyi District in Uganda [98]. A 2008 study in Zambia, measuring absenteeism on the day of the survey (and not through strict surprise visits), finds 31% of doctors, 20% of clinical officers, and 14% of midwives and other clinical staff to be absent.

18 The earlier mentioned study in Ethiopia, using contingent valuation, indicated that to get 80% of health workers to take up a rural position, public sector salaries for nurses and doctors need to increase 57 and 83%, respectively, suggesting that small changes in salaries will have a limited effect on the distribution of health workers. One reason seems to be that in most low-income countries, the discrepancy in amenities between rural and urban areas may be too large to be compensated by salaries alone [84]. A discrete choice experiment study focusing on doctors in Ethiopia finds that doubling wages would increase the share of doctors willing to work in rural areas from 7% to over 50%, while for nurses doubling salaries would increase those willing to work in rural areas from 4 to 27% [42]. Analysis for Tanzania confirms similar strong effects of increasing salary and hardship allowance, with women less responsive to these financial incentives [47]. Focusing on Vietnam, another survey [104] finds that increased pay would be the single most powerful incentive to get health workers willing to locate in rural areas. An older study for Indonesia, Chomitz et al. [24] finds that modest cash incentives can make health workers more likely to work in moderately remote areas but that staffing of very remote areas would be prohibitively expensive.

19 Among benefits, housing appears to be important. The study of doctors in Ethiopia finds that providing high-quality housing would increase the share of doctors willing to work in rural areas to 27% or the equivalent of a wage bonus of 46% [42]. In Tanzania and Malawi, housing is also found to be important. Being able to have a second job is another factor that seems to play an important role [47, 57].

20 Continued access to professional training are found to be important motivating factors for job choice among future health workers in Ethiopia, Kenya, Malawi, South Africa, Tanzania, Thailand and Vietnam [16, 47, 57, 86, 104].

21 See for example Dixit [31] and Le Grand [52] for a discussion on these themes for civil servants in general.

22 For example, on teacher absenteeism [33], on the management and allocation of funds at the school level [79] and on student-learning outcomes [10]. Local monitoring by head teachers as well as communities of beneficiaries have had substantial effects on teacher presence and student-learning outcomes (see [10, 25]).

23 Recent work confirms possible collusion between workers and monitors. A recent study on environmental auditors in Gujarat shows that random allocation of monitors to plants resulted in more reduced pollution than when monitors were chosen by the plants themselves [32].

24 In response to these concerns, Rwanda established a national zero-tolerance for corruption in 2006, which announced a number of measures including regular unannounced evaluations, investigation of suspicious reports, on-site verification of data and validation of receipts of services by a random sample of patients who are recorded. It is unclear to what extent these measures have been followed through, and we know of no evaluation. Bucagu et al. [21] hold a plea for further research to develop systems that improve monitoring but keep the consequences of gaming to a minimum

25 See Bartel et al. [11], Barr and Serneels [9] and Bowles et al. [18] for work on workplace norms outside the health sector.

26 See for example Deci [30], Krepps [49] and Benabou and Tirole [14]. Intrinsic motivation is typically seen as an individual characteristic that is a long term product of both nature and nurture, and that cannot be changed in the short term.

27 Which is more likely to arise in some environments—such as NGOs—than in others because these environments are better suited to overcome agency problems see Glaeser, [41, 75].

28 Burundi (2), DRC (2), Egypt, Philippines (2), Rwanda

29 See de Bruin et al. [29]; Eijkenaar et al. [34]; Kondo et al. [48]; Mendelson et al. [61].

30 The poverty of causal evidence on participatory development initiatives is not restricted to the health sector, as documented by Mansuri and Rao [56]

31 The operational plan sets out a number of focused activities, including a health worker labour market study to take stock of numbers, needs and distribution of health worker and a second study to identify malpractices and unethical behaviour. It plans the development of innovative incentives to attract and retain health personnel, develop an award system for the best performing health professionals and draft a law setting out a career development system for health professionals. Other activities include the plans to develop a certified ethics course, integrating modules of leadership, management and ethics in the training of health professionals [68].
